# Assessing the breadth and multidisciplinarity of the conservation curriculum in the United Kingdom and Australia

**DOI:** 10.1093/biosci/biae059

**Published:** 2024-08-01

**Authors:** Helena Slater, Janet Fisher, George Holmes, Chris Sandbrook, Aidan Keane

**Affiliations:** School of Geosciences at the University of Edinburgh, Edinburgh, Scotland, United Kingdom; School of Geosciences at the University of Edinburgh, Edinburgh, Scotland, United Kingdom; School of Earth and Environment at the University of Leeds, Leeds, England, United Kingdom; Department of Geography, University of Cambridge, Cambridge, England, United Kingdom; School of Geosciences at the University of Edinburgh, Edinburgh, Scotland, United Kingdom

**Keywords:** education, conservation, interdisciplinary science, multidisciplinary, teaching

## Abstract

There have been repeated calls to train conservationists capable of transcending disciplinary boundaries. However, little empirical work has been done to document conservation teaching. We investigate the content taught in conservation higher education across the United Kingdom and Australia. Using data from an online survey and content analysis of module descriptions, we assess the prevalence of subject areas in 146 conservation modules and topics in 368 conservation modules and 62 conservation degrees. Biological sciences subject areas were represented in 92% of the modules, whereas social sciences subject areas only featured in 60% and humanities in 24%. Of the modules teaching biological sciences subject areas, 84% included biological sciences faculty but only 31% of the modules covering social sciences subject areas included faculty from the social sciences. Who teaches matters. The disciplinary expertise in conservation education needs to diversify to train conservationists capable of addressing conservation challenges. This requires institutional changes and support from prominent societies to promote interdisciplinary education.

Although conservation biology has roots in the biological sciences, contemporary conservation science is described as a *metadiscipline*: an interdisciplinary field that draws on a range of academic disciplines and transcends the natural sciences, social sciences, and humanities (Kareiva and Marvier 2012, Gardner 2021). This expansion has coincided with growing recognition that conservation issues are situated in complex social systems and therefore require interdisciplinary responses (Kareiva and Marvier 2012, Bennett et al. 2017a). There are critiques that conservation training has not matched the breadth of knowledge required for research and practice (Newing 2010, Gardner 2021). Despite such critiques, there remains a lack of investigation of whether the disciplinary breadth included in descriptions of conservation is reflected in conservation teaching. Without a clear understanding of what is being taught, we are unable to determine whether future conservationists are receiving the training required to address conservation challenges.

Ineffective and sometimes harmful conservation practices have led to growing appreciation of the need to collaborate across disciplinary boundaries (Bennett et al. 2017a, Claus 2022). It is widely acknowledged that further incorporating diverse academic disciplinary perspectives can help create more just, inclusive, and effective conservation practices and that future conservationists should develop the ability to work with diverse disciplinary perspectives (Sandbrook et al. 2013, Bennett et al. 2017a). Exposing students to knowledge from different fields is an important component of training conservationists who can account for different dimensions of conservation issues (Welch-Devine et al. 2014, Montgomery et al. 2022, Teel et al. 2022). Working across, shifting, and transgressing traditional academic disciplinary silos is a common theme for addressing wicked challenges (Cantor et al. 2015, Lotz-Sisitka et al. 2015).

There are ongoing discussions about what the conservation curriculum should consist of, particularly its breadth and depth. Alongside calls to expand the curriculum to include teaching on the human dimensions of conservation (Jacobson and Duff 1998, Gardner 2021) or social science research methods (Newing 2010) are concerns that broadening the syllabus could result in ineffective jacks of all trades, lacking adequate natural science training (Lidicker 1998). Related debates concern whether we should train interdisciplinary individuals or specialists who are able to work within diverse disciplinary teams (Adams 2007, Dick et al. 2017).

Although there are numerous articles detailing the skills conservation graduates need (Muir and Schwartz 2009, Blickley et al. 2013, Lucas et al. 2017), including interpersonal skills and project management, little empirical work has been done to understand the content of conservation education. Some scholars have investigated specific themes in conservation education, such as interdisciplinarity (Niesenbaum and Lewis 2003) and philosophy (Saltz et al. 2019). These studies provide useful insights but no overview. Other scholars have detailed the content in particular areas of conservation (e.g., tropical conservation; Bonine et al. 2003) or training at postgraduate level (Elliott et al. 2018). The most comprehensive studies of content in UK and Australian conservation degrees have been completed by Van Heezik and Seddon (2005) and Gardner (2021). Both provided an overview of the types of content present in conservation degrees. Still, the studies were focused on a single education level and predominantly relied on syllabus data. There is minimal analysis of the content taught across institutions, countries, and education levels. Overall, despite arguments that we should be equipping the next generations of conservationists with particular knowledge and skills, we have very limited understanding of how they are currently being trained.

Focusing on a sample of conservation higher education teaching within the United Kingdom and Australia, the present study provides a snapshot of conservation teaching and insights into the faculty training conservationists. We use measures of prevalence and breadth to better understand whether the interdisciplinary nature of conservation, as it has been described in the literature (Meine et al. 2006, Kareiva and Marvier 2012, Robert et al. 2017), is reflected in the curriculum. We build on previous reviews (Van Heezik and Seddon 2005, Gardner 2021) by using a combination of data from two methods: an online survey and content analysis of module descriptions.

It is worth noting the diverse ways in which multi- and interdisciplinary terms are used (Klein 2017). Although interdisciplinarity is sometimes used to refer to the provision of different types of disciplinary content (Newing 2010, Gardner 2021), interdisciplinary theory emphasizes *integration* as a key feature that distinguishes interdisciplinarity from multidisciplinarity (Klein 2017). Both multidisciplinarity and interdisciplinarity can also vary in scope (Huutoniemi et al. 2010). Narrow interdisciplinarity integrates fields that are “conceptually close,” whereas broad interdisciplinarity often straddles conceptually distant domains (Huutoniemi et al. 2010, p. 82). We focus on multidisciplinarity, broadly defined as “a conglomeration of disciplinary components” (Huutoniemi et al. 2010, p. 80), by looking at the presence of different subject areas or disciplinary categories rather than their integration (a crucial component of interdisciplinarity but one that is challenging to capture in large-scale comparative work; Lattuca 2001). We discuss results in relation to interdisciplinarity because disciplinary integration is widely acknowledged as important for conservation (Andrade et al. 2014, Pooley et al. 2014) and often builds from multidisciplinarity.

We use *module* to refer to an individual credit-bearing teaching unit that is often taken as a component of a degree. We define *conservation degree* as a degree that contains *conservation* in the title and explicitly aims to teach environmental conservation content. We recognize that conservation is also taught beyond these defined boundaries, but we have deliberately chosen to focus on modules and degrees that explicitly advertise themselves as teaching conservation. This exploratory research was guided by the following questions: What is the prevalence of teaching on different subject areas and how many different disciplinary categories do conservation-specific modules include teaching from? What is the prevalence of teaching on different topics in conservation-specific modules and conservation degrees? Who is involved in conservation-specific teaching and what is their disciplinary expertise? And do the breadth of disciplinary categories and topics taught vary in relation to key module characteristics (country, department, education level and staff disciplinary configuration)?

## Research methods and data analysis

We studied conservation teaching in UK and Australian universities. Both countries have prominent conservation sectors and offer a variety of conservation teaching, across education levels. Although the teaching takes place in different socioecological and historical contexts, the higher education systems of the United Kingdom and Australia share several similarities (Wellings 2015). This includes similar participation rates, uncapped student numbers (Wellings 2015), and the common use of modularization, making the data more easily comparable. At the time of our study, we found 55% of UK and 67% of Australian universities offered conservation degrees.

We investigated the content taught in two units of study: conservation-specific modules and conservation degrees. We investigated content within conservation-specific modules to examine teaching delivered to a broader set of students than just those in conservation degrees. Many conservation researchers enter from single disciplinary contexts such as biology or botany (Montana et al. 2019). For such students, conservation-specific modules may be the only time in which they encounter conservation teaching. As a result, the content taught within conservation modules could play a key role in shaping their understanding of conservation as a field. Alongside conservation-specific modules, we investigated content taught in conservation degrees by collecting data on the core modules in each conservation degree. We focused on core modules because they represent teaching that most, if not all, students will receive.

We chose to classify content using two hierarchical codes: subject areas and topics. The higher-level code, subject areas, refers to broader fields of study that teaching may cover and that typically feature as module classifiers (e.g., ecology). The lower-level code, topics, refers to categories of conservation study that are commonly found in introductory conservation textbooks and that feature in syllabus outlines (e.g., protected areas). Further information on how these codes were developed is provided in the “Data collection methods” section.

### Collating module and degree databases

We created two databases: one for conservation degrees and one for conservation-specific modules. We searched the term *conservation* in online module catalogues and university webpages of each university listed on the Higher Education Statistics Agency (HESA) website for the United Kingdom's 2019–2020 academic year and each university listed on the Australia government website. The degree search results were scanned against predefined criteria (table 1). The databases were also collated for the purposes of a wider research project. As a result, we excluded exclusively online or part-time degree programs, because later stages of the larger research project would require some in-person and full-time teaching. We screened each search result against the criteria (table 1) and, if it qualified for inclusion in the database, it was entered along with the core modules listed in the degree webpage or program specification.

**Table 1.** Search criteria used in conservation degree database procedure.

**Degree inclusion criteria**	**Degree exclusion criteria**
Related to biodiversity/environmental conservation	Degree related to another type of conservation (art/history/architecture)
“conservation” appears in degree title	“conservation” not included in degree title
Degree results in accepted HE qualification (supplement S1)	Short course not resulting in accepted HE qualification (supplement S1)
Includes a minimum of three taught modules	Research degree (MRes that does not include a minimum of three taught modules)
Degree runs for relevant year of study (United Kingdom 2020–2021, Australia 2021–2022)	Information available states degree is not running in the year of study
Degree primarily intended to be taught in person or on campus	Solely distance or online degree program
Offered as a full-time degree	Not solely offered as a part-time degree program

A separate conservation-specific module database was collated by searching for *conservation* in online module catalogues and reviewing degree webpages for modules containing the term. Modules were included in the database if they were related to environmental conservation, offered within a degree that resulted in an accepted qualification (supplement S1), and not exclusively offered in an online or part-time degree program. We collated a degree database containing 126 UK conservation degrees from 57 universities and 26 Australian degrees from 16 universities (supplement S2); 39 universities offered multiple conservation degrees. Our conservation-specific module database contained 460 UK and 108 Australian conservation-specific modules (supplement S3).

### Data collection methods

We used two data collection methods. The first was an online survey instrument, designed to collect data on content covered in conservation modules. The survey was designed as part of a wider research project. One section collected key module information (e.g., department). Another section captured data on subject areas and topics included in the module. Most of the questions were multiple choice (supplement S4). For the prompt “Please select all of the following subject areas that the module includes teaching on,” the respondents were able to select multiple options. The subject options were based on the JACS 3.0 principal subject codes system used by the United Kingdom’s HESA (HESA n.d.). The multiple choice options for topics were created by reviewing 12 conservation textbooks (supplement S5) that frequently appeared as required reading in conservation degrees. The chapters of each textbook were reviewed and summarized to create a list of 19 topics (supplement S6). The respondents were able to select *other* for the multiple-choice questions and to specify their answer in a text box. Each topic option included a description that appeared when the respondent hovered over the topic, and guidance on this was included in the question (supplement S6).

The survey was piloted with three conservation educators at the University of Edinburgh, and minor word changes were implemented following their feedback. The online survey instrument was distributed to known leaders of 334 UK and 90 Australian conservation-specific modules (during June 2020 for the United Kingdom and January 2021 for Australia). The module and degree leaders were identified by searching university webpages and staff directories and by contacting department administration teams. It was not possible to identify all relevant individuals and, as a result, degree leaders were asked to distribute the survey to their module leaders. We received survey responses for 117 UK and 29 Australian conservation-specific modules (corresponding to 25% of the UK and 27% of the Australian conservation-specific modules in our database).

We used a second data collection method to collect information on conservation-specific modules and core modules that we did not receive survey responses for. We reviewed online module descriptions and used a predefined protocol to code the information available (supplement S7). The first author and two research assistants systematically searched university webpages and module catalogues for publicly accessible module descriptions. We first searched each university website for a module catalogue, and where catalogues were unavailable, we searched university webpages for the module name. If a module description was still not found, we completed a Google search including the university name, the module title, and the module code (if known). To be included, the description needed to include a section on the content taught in the module and a section on the learning outcomes or skills the module aimed to develop. Each description that met this criterion was reviewed against a content analysis protocol. This protocol used the same topic options as the survey to record where a module description mentioned a topic or defined key terms associated with the topic description. The team reviewed a sample of each other's coding, and any disagreements between the reviewers were discussed until there was a consensus.

**Figure 1.**
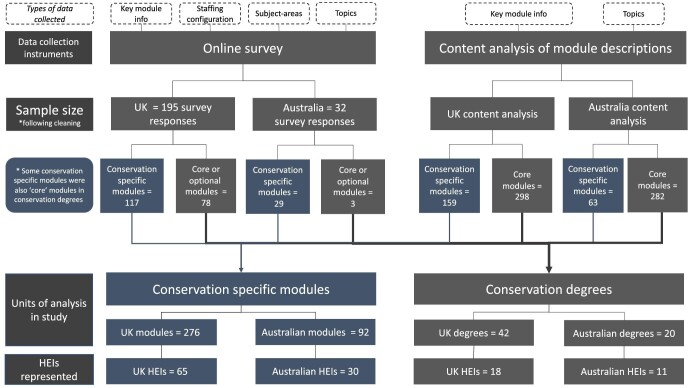
Summary of data collection and final units of study.

We did not collect data on subject areas during the content analysis of descriptions. While developing the protocol and reviewing example descriptions, we decided it would be challenging to classify the subject areas covered (e.g., law) on the basis of the descriptions available. As a result, we reviewed module descriptions for just lower-level codes (topics). Through content analysis, we collected data on an additional 159 UK and 63 Australian conservation specific modules (figure 1).

In total, we collected survey or content analysis data on 276 UK conservation-specific modules (from 65 UK universities) and 92 Australian conservation-specific modules (from 30 Australian universities). Using a combination of survey response and content analysis data, we collected data on all core modules in a total of 42 UK and 20 Australian conservation degrees (from 18 UK universities and 11 Australian universities). All of the universities represented in the core module data were represented in the conservation-specific module sample.

To check the veracity of data collected through the content analysis protocol, we compared the topics recorded using the survey instrument and content analysis method for a sample of 20 conservation-specific modules. The survey responses included a higher number of topics than those captured by reviewing the online module descriptions (survey mean = 13.55, content analysis mean = 8.25; supplement S8).

We decided to use a combination of data collected through these methods to provide a more comprehensive overview of the modules in our database. The data on most core modules, which we aggregate into conservation degrees, were sourced through the content analysis method. We recognize there are some systematic biases in using data sourced through online module descriptions and the content analysis method may underrepresent the breadth of topics covered in a module. Where possible, we present the results split by data source (survey or content analysis). Extra supplementary analysis show differences in the prevalence of topics recorded through these two methods.

### Data analysis

Prior to analysis, survey response data were cleaned to remove entries that did not meet the study criteria. For analysis, we only included degrees where survey or content analysis data were collected for all core modules. We classified the subject areas into five disciplinary categories on the basis of the UK HESA groupings (supplement S9). As an indicator of breadth, we calculated the number of different disciplinary categories represented in each conservation-specific module. We used descriptive statistics to investigate the prevalence of content and staff disciplinary expertise. Data analysis and visualizations were completed in R 4.2.1 (R Core Team 2022). The data are available in Edinburgh DataShare (see data availability statement).

To investigate any differences in the breadth of teaching on different disciplinary categories or topics in relation to module characteristics, we fitted two mixed effect models (one for disciplinary categories and another for topics) using the lme4 package (Bates et al. 2015). We used solely survey response data for conservation-specific modules to fit both models because staffing information was limited in online module descriptions.

To fit both models, we created a binary response variable that indicated whether a disciplinary category or topic was covered in the conservation-specific module (1, *yes*; 0, *no*). A unique module code was assigned to each module and included as a random effect in both models. Four module characteristics were included as explanatory variables: department (natural sciences, interdisciplinary, social sciences), exclusive to postgraduate students (1, *yes*; 0, *no*), academic staff from different disciplinary categories (1, *yes*; 0, *no*) and country (United Kingdom, Australia).

Most of the survey respondents selected a department option that best described where their module was housed. Any modules in which *other* was selected for the department were sorted into categories by reviewing the text provided and screening the department descriptions for each case. The survey respondents were asked to specify the disciplinary categories of academic staff involved in the module. We sorted *other* survey responses into staff disciplinary categories by reviewing any text provided (supplement S10). The modules were classed as including academic staff from different disciplinary categories if they selected at least two of the multiple-choice options.

All explanatory variables were fitted as fixed effects in both models (supplement S11). In the disciplinary categories model, we fitted the disciplinary category variable as a fixed effect to investigate any significant differences in the prevalence of disciplinary categories while accounting for module characteristics. For the topics model, topic was included as a random effect as we were interested in the breadth of topics covered rather than testing for difference in prevalence of topics. To visualize differences, we plotted the estimates of the fixed effect terms against baseline reference levels.

As a result of differences in the data available through the content analysis method compared with the survey instrument, the following results report analysis using different subsets of data. Analyses relating to subject areas and staff configuration uses solely survey response data for conservation-specific modules. In the figure captions, we detail the data sources that contributed to the analysis and the relevant sample sizes.

## Results

### Subject areas taught in conservation-specific modules

**Figure 2.**
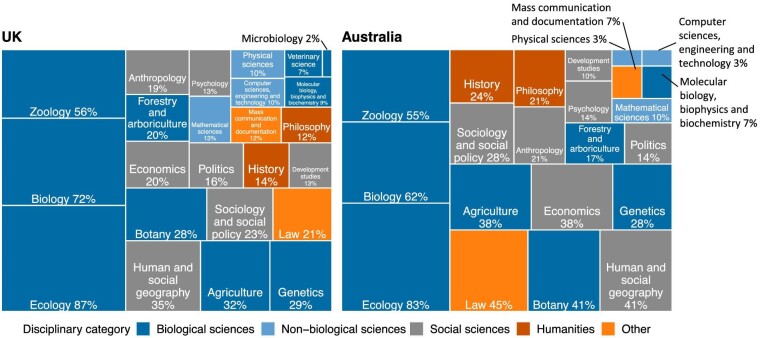
Tree map of subject areas covered in conservation-specific modules. This analysis used solely survey response data (UK module, n = 117; Australia module, n = 29). The size of the shapes corresponds to the frequency of modules covering a subject area.

In the 146 conservation-specific modules analyzed, 24 subject areas were represented from across five disciplinary categories: biological sciences, nonbiological sciences, social sciences, humanities, and other (figure 2). Ecology was present in 86% of conservation-specific modules, biology in 70%, and zoology in 56%. In our analysis of Likert items on interdisciplinarity in conservation training, designed to collect data for a separate study within the wider research project, 54% of the UK and 48% of the Australian respondents disagreed with the statement “The natural sciences should be the primary focus of conservation training” (supplement S12).

Of the seven subject areas classed as social sciences in our study, some featured more frequently than others. Human and social geography was the most prevalent social science subject area in both the UK (35%) and the Australian (41%) conservation modules. Anthropology was covered in 19% of the conservation-specific modules, psychology in 13%, and politics in 16%. Law was more frequently taught in the Australian modules (45%) than those in the United Kingdom (21%). Of the humanities subject areas in our categorization, history was present in 16% of the conservation-specific modules and philosophy in 14%.

On average, the UK conservation-specific modules covered 5.7 subject areas, and the Australian modules covered 6.1 subject areas. However, there was a wide variation in the number of subject areas covered in the conservation modules surveyed (supplement S13).

**Figure 3.**
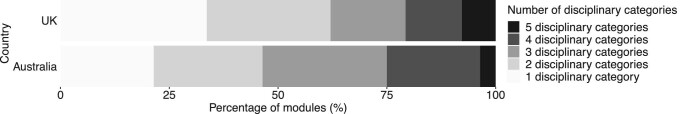
The percentage of modules covering subject areas from one or more disciplinary categories. The analysis used solely survey response data for conservation-specific modules. One UK and one Australian module were excluded because they solely selected other for the subject areas covered (UK module, n = 116; Australian module, n = 28).

Most of the conservation-specific modules included teaching on subject areas from two or more disciplinary categories (figure 3). A higher proportion of the Australian conservation modules covered subject areas from two or more disciplinary categories. Of the UK conservation modules, 29% included subject areas solely from the biological sciences, compared with 21% of the Australian modules.

### Topics taught in conservation-specific modules and conservation degrees

**Figure 4.**
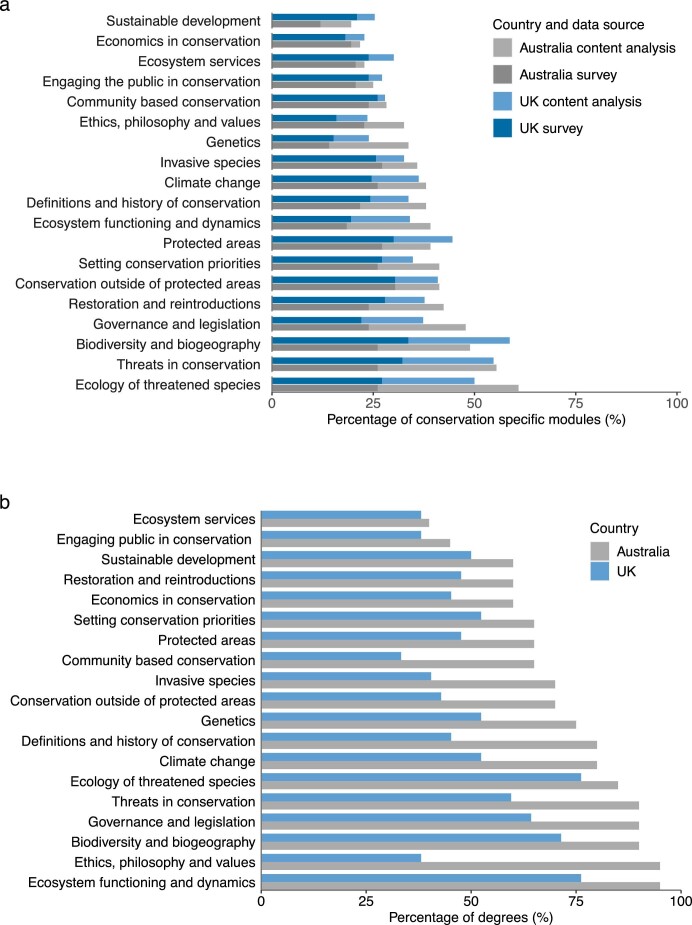
The prevalence of topics in conservation-specific modules (a) and conservation degrees (b). Panel (a) uses a combination of survey and content analysis data for conservation-specific modules (UK module, n = 276; Australian module, n = 92). Panel (b) uses a combination of survey and content analysis data for core modules in each conservation degree. The bars represent the percentage of degrees that include at least one core module covering a given topic (UK degree, n = 42; Australian degree, n = 20). Panel (b) is not split by data source as core modules, made up of both survey and content analysis data, were aggregated into degrees.

Using a combination of survey and content analysis data, the most prevalent topics in the conservation-specific modules were the ecology of threatened species, threats in conservation, and biodiversity and biogeography (figure 4). Less frequently covered topics included sustainable development, economics in conservation, and ecosystem services.

Some differences were found between the prevalence of the topics recorded using the two data collection methods (supplement S14 and figure 4a). UK conservation-specific modules covered a mean of 6.76 topics, and Australian conservation-specific modules a mean of 7.11 topics. There was some heterogeneity in the breadth of topics covered, with some modules selecting 1 topic, whereas others selected all 19 topics (supplement S15).

Most of the conservation degrees included at least one core module covering ecosystem functioning (United Kingdom, 76%; Australia, 95%) or biodiversity and biogeography (United Kingdom, 71%; Australia, 90%). The topics absent from the core offering of most of the degrees included ecosystem services and engaging the public in conservation. Ethics, philosophy, and values in conservation were taught in the majority (95%) of the Australian degrees but were only present in the core offering of 38% of the UK conservation degrees. Further supplementary analyses showed differences in the prevalence of the topics between undergraduate and postgraduate degrees (supplement S16).

### Staffing configuration in conservation-specific modules

Most of the conservation-specific modules surveyed were housed in natural science departments (United Kingdom, 66%; Australia, 62%); 14% were housed in a social sciences department, and 21% were in an interdisciplinary department (supplement S17). Most (77%) of the modules included academic staff from the biological sciences, 20% included faculty from the social sciences, and less than 2% included an academic member of staff from the humanities (supplement S18). Most conservation-specific modules were taught by two or more members of staff (United Kingdom, 74%; Australia, 90%). Of the 134 conservation-specific modules covering biological sciences subject areas, 84% included academic staff from the biological sciences, and just 16% did not include faculty from the biological sciences. Of the modules covering social sciences subject areas, 69% did not include an academic staff member from the social sciences (supplement S19). Of the 24% of the modules covering the subject areas classed in the humanities, just one module included faculty from the humanities.

### Breadth of content taught in relation to module characteristics

**Figure 5.**
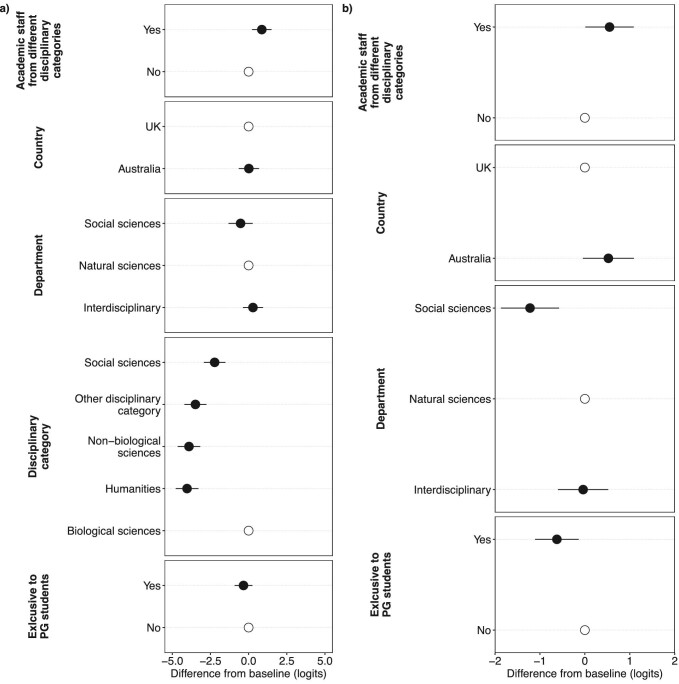
Links between the breadth of content taught and the module characteristics. (a) The breadth of disciplinary categories taught. (b) The breadth of topics taught. In both panels, the unfilled circles indicate the baseline reference level against which the other levels are compared. The filled circles represent the average difference from the baseline level (in logits), and the error bars represent 95% confidence intervals.

Our model for disciplinary categories revealed that, when controlling for module characteristics, nonbiological sciences, humanities, and social sciences subject areas were all taught significantly less than biological sciences (figure 5). We found a significant difference in the breadth of disciplinary categories taught in relation to the presence of staff from different disciplinary groups. The modules with academic staff from different disciplinary categories were associated with covering a broader range of disciplinary categories than the modules with staff from one disciplinary group. We did not observe any significant differences in the breadth of disciplinary categories taught for the remaining explanatory variables (department, exclusivity to postgraduates, country).

Some differences in the breadth of topics taught were observed in relation to the module characteristics. The modules exclusive to postgraduates were associated with covering a lower breadth of topics than the modules open to undergraduates. The modules in social sciences departments covered, on average, a lower number of topics than the modules in natural sciences departments.

## Discussion

The study provides the most extensive review of conservation teaching content to date, presenting data on 368 conservation-specific modules and 62 conservation degrees. Most of the conservation modules displayed some degree of breadth, covering subject areas from two or more disciplinary categories. Still, biology-oriented content appeared to be the most prevalent. Some subject areas and topics typically associated with the social dimensions of conservation were poorly represented. If future conservationists are to untangle messy conservation challenges that require interdisciplinary thinking, the provision of non-natural-science teaching needs to increase. This will require diversifying the disciplines involved in conservation training and support from conservation societies to promote interdisciplinary education.

### Prevalence and breadth of subject areas

Our analysis provides empirical evidence that a diverse range of subject areas are represented in conservation teaching. Most of the conservation-specific modules included teaching on two or more disciplinary categories, highlighting the breadth of conservation as a field. The conservation modules covering subject areas solely from the biological sciences were in the minority (for the United Kingdom, 29%; for Australia, 21%). These findings are somewhat encouraging. They indicate that some conservation students are already receiving some degree of multidisciplinary teaching.

However, the only subject areas to feature in over half of the sample were classed as biological sciences. Ecology, biology, and zoology were the most frequently taught subject areas across the UK and Australian conservation-specific modules. This supports previous research that has suggested that, despite some heterogeneity, most conservation teaching remains biology-oriented (Saberwal et al. 1996, Gardner 2021). The prominence of biological science content is likely to relate to the disciplinary background and expertise of conservation educators. Our model indicates a difference in the breadth of disciplinary categories taught when comparing modules with a mix of staff from different disciplinary categories with those with academic staff from a single disciplinary group. The continued prevalence of biological science content may relate to dominant perceptions of what conservation is and expectations of what should be taught. Some UK conservation degrees are accredited by the Chartered Institute of Ecology and Environmental Management, which places an emphasis on training ecologists (CIEEM 2021) rather than interdisciplinary thinkers (Andrade et al. 2014) or agile conservationists (Welch-Devine et al. 2014). In the context of a marketized higher education sector, where students are often characterized as consumers (Hemsley‐Brown and Oplatka 2006), the prevalence of biological sciences may also relate to student expectations of what should be taught. Diversifying the disciplinary perspectives included in teaching could help future conservationists to understand and practice conservation as an interdisciplinary endeavor.

Despite repeated calls to increase social science training (Newing 2010, Teel et al. 2022) and teaching on the human dimensions of conservation (Cannon et al. 1996, Jacobson and Duff 1998), our results indicate that social science and humanities subject areas remain underrepresented in comparison to biological sciences subject areas. Psychology, sociology, and anthropology all featured in less than a quarter of the conservation modules surveyed, despite demand for greater social science training among early career conservationists (Fisher et al. 2009, Archer et al. 2022). The social sciences add significant value to conservation (Bennett 2016, Bennett et al. 2017a, 2017b, Selinske et al. 2018) and are necessary for training students who can achieve a holistic understanding of conservation issues (Teel et al. 2022).

Moreover, the humanities make important contributions to conservation (Holmes et al. 2021). For instance, philosophy can help students to unpick the philosophical tensions that underlie a value-laden field (Saltz et al. 2019). History provides crucial insights into the context and underlying causes of contemporary conservation issues (Drayton 2000, Pooley et al. 2023). Incorporating further teaching on social sciences and humanities subject areas, while fostering reflexivity, could help to build more ethical conservation practices (Brittain et al. 2020, Montana et al. 2020, Holmes et al. 2021, Pienkowski et al. 2023). A limitation of this study is that we did not measure the depth of training on different content. Future research into the relative time spent on different types of content could be a fruitful avenue to explore.

### Topic prevalence in conservation modules and degrees

The prevalence of teaching on the ecology of threatened species and threats in conservation was somewhat expected; these topics have consistently featured in conservation curricula (White et al. 2000, Van Heezik and Seddon 2005). We found topics more typically associated with the social aspects of conservation were less frequently covered. For instance, engaging the public in conservation and economics in conservation were among the least prevalent topics. The topics broadly categorized under the label of human dimensions were similarly less prevalent than some biology-oriented topics in previous studies of conservation education (Van Heezik and Seddon 2005, Dayer and Mengak 2020). With growing recognition that conservation challenges require interdisciplinary approaches (Pooley et al. 2014), there is room for professional conservation societies to emphasize the importance of teaching on different dimensions of conservation. Currently, the Society for Conservation Biology's recommended guidelines for conservation literacy include the primary principle that “conservation is based on key concepts in taxonomy, ecology, genetics, geography, and evolution.” (Trombulak et al. 2004, p 1188). These guidelines could be updated to reflect the expansion of conservation science and to emphasize the need for teaching on the social dimensions of conservation. It should be noted that our analysis was limited to topics identified through reviewing conservation textbooks. Further research could look at an expanded set of topics and explore how topics are framed in conservation teaching.

Our analysis revealed some differences in the prevalence and breadth of topics covered when varying module characteristics. Almost all of the Australian degrees reviewed included a core module covering ethics, philosophy, and values in conservation. In contrast, less than half of the UK conservation degrees included a core module covering this topic. There may be several reasons for such differences. One methodological explanation may be that Australian module descriptions typically included detailed information, and therefore, the topics were captured to a greater degree than those in the United Kingdom. The differences in socioecological and geographical contexts are also likely to shape the prominence of certain topics. For instance, although invasive species are problematic in both countries, the associated challenges are highlighted to a greater degree in Australia (Hoffmann and Broadhurst 2016). Ethics, philosophy, and values in conservation may be more commonly taught in Australian modules than those in the United Kingdom, given that Australian conservation practice takes place in relation to the land ownership and rights of Australian Indigenous peoples (Leiper et al. 2018). However, teaching on history and ethics should also be a common component of conservation training in the United Kingdom, given Britain's role in colonialism and its lasting impact on conservation (Adams and Mulligan 2002). The breadth of topics covered in Australian degrees compared with the United Kingdom could also relate to differences in education infrastructures. Our analysis is limited to teaching in the United Kingdom and Australia. Further investigation is required to determine whether these trends are reflected across different geographic contexts.

### Disciplinary expertise involved in conservation-specific teaching

Most conservation-specific modules included academic staff from the biological sciences, whereas the presence of staff from the social sciences and humanities was rare. Of the modules covering social science subject areas, 69% did not include an academic from the social sciences. In contrast, just 16% of the modules covering biological sciences subject areas did not include a staff member from the biological sciences. There are multiple examples of expertise from the social sciences being included too late or undervalued in conservation research and practice (Martin 2020, Claus 2022). Who teaches matters. The disciplinary expertise involved in conservation training needs diversifying to address the imbalance among the natural sciences, social sciences, and humanities.

The barriers to interdisciplinary research and teaching are well documented (Fox et al. 2006, Andrade et al. 2014, Dick et al. 2017). Persistent institutional obstacles hinder interdisciplinary collaboration (Brewer 1999, Lindvig et al. 2019). Considerable institutional level changes are required to encourage interdisciplinary education and to support educators in overcoming challenges associated with interdisciplinary teaching (Shapiro and Dempsey 2008, Keeley and Benton-Short 2020). One recommendation may be for those trained within the social sciences and humanities to codesign modules with conservationists from a natural science background. Such efforts would require sufficient resources to allow educators to navigate their own disciplinary differences and communication issues (Shibley 2006). Another option would be to incentivize educators to teach beyond their host department or for conservation degrees to be established in explicitly interdisciplinary departments that make a clear mission to transcend disciplinary silos.

### Conclusions

This study provides a snapshot of the content covered in conservation higher education across the United Kingdom and Australia. We found a diverse range of subject areas and topics represented in conservation teaching. Still, biological sciences content appeared the most prevalent. The imbalance between the natural sciences, social sciences, and humanities in conservation training needs to be addressed. Further mainstreaming the social sciences and humanities into conservation training is an important step toward addressing the knowledge asymmetry common in conservation (Claus 2022) and for building inclusive conservation practices. Incorporating non-natural-science expertise into teaching could help students to develop a more holistic understanding of the multiple dimensions of conservation challenges. If conservation science is an inherently interdisciplinary field, then this should be fully reflected in conservation training. Conservation educators can make steps to collaborate with expertise beyond their own departments, but significant institutional changes are required and prominent societies should actively promote interdisciplinary education.

## Supplementary Material

biae059_Supplemental_Files

## Data Availability

Data needed to support the analyses have been deposited in the Edinburgh DataShare digital repository at https://doi.org/10.7488/ds/7739.

## Author Biography

Helena Slater (helena.slater@ed.ac.uk) Aidan Keane, and Janet Fisher are all affiliated with the School of Geosciences at the University of Edinburgh, in Edinburgh, Scotland, in the United Kingdom. George Holmes is affiliated with the School of Earth and Environment at the University of Leeds, in Leeds, England, in the United Kingdom. Chris Sandbrook is affiliated with the Department of Geography at the University of Cambridge, in Cambridge, England, in the United Kingdom.

## References

[bib2] Adams WM . 2007. Thinking like a human: Social science and the two cultures problem. Oryx 41: 275–276.

[bib1] Adams W, Mulligan M. 2002. Decolonizing Nature: Strategies for Conservation in a Post-Colonial Era. Routledge.

[bib3] Andrade K, Corbin C, Diver S, Eitzel MV, Williamson J, Brashares J, Fortmann L. 2014. Finding your way in the interdisciplinary forest: Notes on educating future conservation practitioners. Biodiversity and Conservation 23: 3405–3423.

[bib4] Archer LJ, Müller HS, Jones LP, Ma H, Gleave RA, Da Silva Cerqueira A, McMurdo Hamilton T, Shennan-Farpón Y. 2022. Towards fairer conservation: Perspectives and ideas from early-career researchers. People and Nature 4: 612–626.

[bib5] Bates D, Mächler M, Bolker B, Walker S. 2015. Fitting linear mixed-effects models using lme4. Journal of Statistical Software 67: 1–48.

[bib6] Bennett NJ . 2016. Using perceptions as evidence to improve conservation and environmental management. Conservation Biology 30: 582–592. 26801337 10.1111/cobi.12681

[bib7] Bennett NJ et al. 2017a. Mainstreaming the social sciences in conservation. Conservation Biology 31: 56–66. 27334309 10.1111/cobi.12788

[bib8] Bennett NJ et al. 2017b. Conservation social science: Understanding and integrating human dimensions to improve conservation. Biological Conservation 205: 93–108.

[bib9] Blickley JL, Deiner K, Garbach K, Lacher I, Meek MH, Porensky LM, Wilkerson ML, Winford EM, Schwartz MW. 2013. Graduate student's guide to necessary skills for nonacademic conservation careers. Conservation Biology 27: 24–34. 23140555 10.1111/j.1523-1739.2012.01956.x

[bib10] Bonine K, Reid J, Dalzen R. 2003. Training and education for Tropical conservation. Conservation Biology 17: 1209–1218.

[bib11] Brewer GD . 1999. The challenges of interdisciplinarity. Policy Sciences 32: 327–337.

[bib12] Brittain S et al. 2020. Ethical considerations when conservation research involves people. Conservation Biology 34: 925–933. 31953971 10.1111/cobi.13464

[bib13] Cannon JR, Dietz JM, Dietz LA. 1996. Training conservation biologists in Human interaction skills. Conservation Biology 10: 1277–1282.

[bib14] Cantor A, DeLauer V, Martin D, Rogan J. 2015. Training interdisciplinary “wicked problem” solvers: Applying lessons from HERO in community-based research experiences for undergraduates. Journal of Geography in Higher Education 39: 407–419.

[bib15] [CIEEM] Chartered Institute of Ecology and Environmental Management . 2021. Higher Education Degree Accreditation Handbook: A Guide to CIEEM Accreditation for Undergraduate Degree Programmes and Named Pathways through a Degree. CIEMM.

[bib16] Claus CA . 2022. Conservation social scientists in transnational institutions: Negotiating hierarchies of expertise. Conservation and Society 20: 268–277.

[bib17] Dayer AA, Mengak LF. 2020. Human Dimensions of Wildlife Human dimensions in undergraduate fisheries and wildlife degree programs in United States. Human Dimensions of Wildlife 25: 478–488.

[bib18] Dick M, Rous AM, Nguyen VM, Cooke SJ. 2017. Necessary but challenging: Multiple disciplinary approaches to solving conservation problems. Facets 1: 67–82.

[bib19] Drayton RH . 2000. Nature's Government: Science, Imperial Britain, and the “Improvement” of the World. Yale University Press.

[bib20] Elliott L, Ryan M, Wyborn C. 2018. Global patterns in conservation capacity development. Biological Conservation 221: 261–269.

[bib21] Fisher B, Balmford A, Green RE, Trevelyan R. 2009. Conservation science training: The need for an extra dimension. Oryx 43: 361–363.

[bib22] Fox HE, Christian C, Nordby JC, Pergams ORW, Peterson GD, Pyke CR. 2006. Perceived barriers to integrating social science and conservation. Conservation Biology 20: 1817–1820. 17181819 10.1111/j.1523-1739.2006.00598.x

[bib23] Gardner CJ . 2021. Not teaching what we practice: Undergraduate conservation training at UK universities lacks interdisciplinarity. Environmental Conservation 48: 65–70.

[bib24] Hemsley-Brown J, Oplatka I. 2006. Universities in a competitive global marketplace: A systematic review of the literature on higher education marketing. International Journal of Public Sector Management 19: 316–338.

[bib25] [HESA] Higher Education Statistics Agency . n.d. JACS 3.0: Principal Subject Codes. HESA. www.hesa.ac.uk/support/documentation/jacs/jacs3-principal.

[bib26] Hoffmann BD, Broadhurst LM. 2016. The economic cost of managing invasive species in Australia. NeoBiota 31: 1–18.

[bib27] Holmes G, Carruthers-Jones J, Huggan G, de Smalen ER, Ritson K, Šimková P. 2021. Mainstreaming the humanities in conservation. Conservation Biology 36: e13824. 34425030 10.1111/cobi.13824

[bib28] Huutoniemi K, Klein JT, Bruun H, Hukkinen J. 2010. Analyzing interdisciplinarity: Typology and indicators. Research Policy 39: 79–88.

[bib29] Jacobson SK, Duff MD. 1998. Training idiot savants: The lack of Human dimensions in conservation biology. Conservation Biology 12: 263–267.

[bib30] Kareiva P, Marvier M. 2012. What is conservation science? BioScience 62: 962–969.

[bib31] Keeley M, Benton-Short L. 2020. Holding complexity: Lessons from team-teaching an interdisciplinary collegiate course on urban sustainability. Social Sciences 9: 1–18.

[bib32] Klein JT . 2017. Typologies of interdisciplinarity: The boundary work of definition. Pages 21–34 in Frodeman R, ed. The Oxford Handbook of Interdisciplinarity 2nd ed. Oxford University Press.

[bib33] Lattuca LR . 2001. Creating Interdisciplinarity: Interdisciplinary Research and Teaching among College and University Faculty. Vanderbilt University Press.

[bib34] Leiper I, Zander KK, Robinson CJ, Carwadine J, Moggridge BJ, Garnett ST. 2018. Quantifying current and potential contributions of Australian indigenous peoples to threatened species management: Indigenous peoples and threatened species. Conservation Biology 32: 1038–1047. 30035336 10.1111/cobi.13178

[bib35] Lidicker WZJ . 1998. Revisiting the human dimension in conservation biology. Conservation Biology 12: 1170–1172.

[bib36] Lindvig K, Lyall C, Meagher LR. 2019. Creating interdisciplinary education within monodisciplinary structures: The art of managing interstitiality. Studies in Higher Education 44: 347–360.

[bib37] Lotz-Sisitka H, Wals AEJ, Kronlid D, McGarry D. 2015. Transformative, transgressive social learning: Rethinking higher education pedagogy in times of systemic global dysfunction. Current Opinion in Environmental Sustainability 16: 73–80.

[bib38] Lucas J, Gora E, Alonso A. 2017. A view of the global conservation job market and how to succeed in it. Conservation Biology 31: 1223–1231. 28464283 10.1111/cobi.12949

[bib39] Martin VY . 2020. Four common problems In environmental social research undertaken by natural scientists. BioScience 70: 13–16.

[bib40] Meine C, Soulé M, Noss RF. 2006. “A mission-driven discipline”: The growth of conservation biology. Conservation Biology 20: 631–651. 16909546 10.1111/j.1523-1739.2006.00449.x

[bib42] Montana J, Sandbrook C, Robertson E, Ryan M. 2019. Revealing research preferences in conservation science. Oryx 55: 404–411.

[bib41] Montana J, Elliott L, Ryan M, Wyborn C. 2020. The need for improved reflexivity in conservation science. Environmental Conservation 47: 217–219.

[bib43] Montgomery RA, Pointer AM, Jingo S, Kasozi H, Ogada M, Mudumba T. 2022. Integrating social justice into higher education conservation science. BioScience 72: 549–559. 35677291 10.1093/biosci/biac008 PMC9169897

[bib44] Muir MJ, Schwartz MW. 2009. Academic research training for a nonacademic workplace: A case study of graduate student alumni who work in conservation. Conservation Biology 23: 1357–1368. 19758390 10.1111/j.1523-1739.2009.01325.x

[bib45] Newing H . 2010. Interdisciplinary training in environmental conservation: Definitions, progress and future directions. Environmental Conservation 37: 410–418.

[bib46] Niesenbaum RA, Lewis T. 2003. Ghettoization in conservation biology: How interdisciplinary is our teaching? Conservation Biology 17: 6–10.

[bib47] Pienkowski T et al. 2023. Recognizing reflexivity among conservation practitioners. Conservation Biology 37: e14022. 36285608 10.1111/cobi.14022

[bib49] Pooley SP, Mendelsohn JA, Milner-Gulland EJ. 2014. Hunting down the chimera of multiple disciplinarity in conservation science. Conservation Biology 28: 22–32. 24299167 10.1111/cobi.12183 PMC4232892

[bib48] Pooley SP, Hill C, Linnell J. 2023. How histories shape interactions. Pages 68–73 in [IUCN] International Union for Conservation of Nature. IUCN SSC Guidelines on human–wildlife Conflict and Coexistence. IUCN.

[bib50] R Core Team . 2022. R: A Language and Environment for Statistical Computing. R Foundation for Statistical Computing.

[bib51] Robert A et al. 2017. Fixism and conservation science. Conservation Biology 31: 781–788. 27943401 10.1111/cobi.12876

[bib52] Saberwal VK, Kotharit A. 1996. The human dimension in conservation biology curricula in developing countries. Conservation Biology 10: 1328–1331.

[bib53] Saltz D, Justus J, Huffaker B. 2019. The crucial but underrepresented role of philosophy in conservation science curricula. Conservation Biology 33: 217–220. 29947116 10.1111/cobi.13162

[bib54] Sandbrook C, Adams WM, Büscher B, Vira B. 2013. Social research and biodiversity conservation. Conservation Biology 27: 1487–1490. 24033825 10.1111/cobi.12141

[bib55] Selinske MJ, Garrard GE, Bekessy SA, Gordon A, Kusmanoff AM, Fidler F. 2018. Revisiting the promise of conservation psychology. Conservation Biology 32: 1464–1468. 29604116 10.1111/cobi.13106

[bib56] Shapiro EJ, Dempsey CJ. 2008. Conflict resolution in team teaching: A case study in interdisciplinary teaching. College Teaching 56: 157–162.

[bib57] Shibley IA . 2006. Interdisciplinary team teaching: Negotiating pedagogical differences. College Teaching 54: 271–274.

[bib59] Teel TL, Bruyere B, Dayer A, Stoner KE, Bishop C, Bruskotter J, Freeman S, Newmark J, Jager C, Manfredo MJ. 2022. Reenvisioning the university education needs of wildlife conservation professionals in the United States. Conservation Science and Practice 4: e610.

[bib60] Trombulak SC, Omland KS, Robinson JA, Lusk JJ, Fleischner TL, Brown G, Domroese M. 2004. Principles of conservation biology: Recommended guidelines for conservation literacy from the education committee of the society for conservation biology. Conservation Biology 18: 1180–1190.

[bib61] Van Heezik Y, Seddon PJ. 2005. Structure and content of graduate wildlife management and conservation biology programs: An international perspective. Conservation Biology 19: 7–14.

[bib62] Welch-Devine M, Hardy D, Brosius JP, Heynen N. 2014. A pedagogical model for integrative training in conservation and sustainability. Ecology and Society 19: 26269578.

[bib63] Wellings P . 2015. The architecture and the plumbing: What features do the higher education systems in the UK and Australia have in common? Perspectives: Policy and Practice in Higher Education 19: 71–78.

[bib64] White R, Fleischner TL, Trombulak SC. 2000. The status of undergraduate education in conservation biology. Conservation Biology 32: 73.

