# Supplementary material for: Assessing the breadth and multidisciplinarity of the conservation curriculum in the United Kingdom and Australia
Source: Bioscience. 2024 Aug 1;74(9):652–62. doi: 10.1093/biosci/biae059 (PMC11480662; doi:10.1093/biosci/biae059)
Supplement: biae059_Supplemental_Files [file biae059_supplemental_files.zip › Appendix_S8.pdf]

Country	Module name	Comparison of topics recorded using survey vs content analysis method					
		Number of topics recorded in survey response	Number of topics recorded in content analysis	Number of identical topics recorded in survey response and content analysis	Number of topics recorded in survey response but missing from content analysis	Number of topics recorded in content analysis but missing from survey response	Percentage of topics recorded in content analysis that matched the topics recorded in the survey response
UK	Module A	15	9	8	7	1	53%
UK	Module B	10	7	6	4	1	60%
UK	Module C	6	2	1	5	1	17%
UK	Module D	8	3	2	6	1	25%
UK	Module E	19	12	12	7	0	63%
UK	Module F	16	9	9	7	0	56%
UK	Module G	9	10	7	2	3	78%
UK	Module H	17	10	10	7	0	59%
UK	Module I	16	9	9	7	0	56%
UK	Module J	13	4	3	10	1	23%
Australia	Module K	18	12	12	6	0	67%
Australia	Module L	12	6	4	8	2	33%
Australia	Module M	17	16	14	3	2	82%
Australia	Module N	4	4	2	2	2	50%
Australia	Module O	13	9	8	5	1	62%
Australia	Module P	16	7	7	9	0	44%
Australia	Module Q	13	7	6	7	1	46%
Australia	Module R	19	9	9	10	0	47%
Australia	Module S	19	12	12	7	0	63%
Australia	Module T	11	8	6	7	2	55%

Average percentage of identical topics captured in content analysis and survey response data	52%
