# Supplementary material for: Assessing the breadth and multidisciplinarity of the conservation curriculum in the United Kingdom and Australia
Source: Bioscience. 2024 Aug 1;74(9):652–62. doi: 10.1093/biosci/biae059 (PMC11480662; doi:10.1093/biosci/biae059)
Supplement: biae059_Supplemental_Files [file biae059_supplemental_files.zip › Supplementary_Materials_Bioscience.docx]

Table of Contents

[Appendix S1. Table of accepted higher education qualifications for collating the conservation degree database. 2](#_Toc137737556)

[Appendix S2. Database of conservation specific degrees. 2](#_Toc137737557)

[Appendix S3. Database of conservation specific modules. 3](#_Toc137737558)

[Appendix S4. Copy of online survey instrument. 3](#_Toc137737559)

[Appendix S5. List of conservation textbooks reviewed to create a list of conservation-specific topics. 3](#_Toc137737560)

[Appendix S6. List of topics and short descriptions for each. 4](#_Toc137737561)

[Appendix S7. Content analysis protocol for online module descriptions and example of coding. 5](#_Toc137737562)

[Appendix S9: Classification of subject-areas into disciplinary categories 6](#_Toc137737563)

[Appendix S10: Sorting of ‘other’ disciplinary staff responses into disciplinary categories 7](#_Toc137737564)

[Appendix S11. Code used to fit generalized linear mixed effect models in RStudio. 8](#_Toc137737565)

[Appendix S12: Likert responses to interdisciplinary statements in final section of the online survey instrument. 9](#_Toc137737566)

[Appendix S13. Boxplot of the frequency of subject areas covered by conservation specific modules. 11](#_Toc137737567)

[Appendix 14: Topic frequencies split by survey response and content analysis data. 11](#_Toc137737568)

[Appendix S15. Boxplot of number of topics covered in conservation specific modules, grouped by country and education level. 12](#_Toc137737569)

[Appendix S16. Conservation degrees including at least 1 core module on conservation topics. 13](#_Toc137737570)

[Appendix S17: Percentage of conservation specific modules belonging to each type of department. 14](#_Toc137737571)

[Appendix S18: Percentage of conservation specific modules including staff from each disciplinary category. 14](#_Toc137737572)

[Appendix S19. Percentage of modules covering biological or social sciences, and whether they include the corresponding disciplinary staff expertise. 15](#_Toc137737573)

# Appendix S1. Table of accepted higher education qualifications for collating the conservation degree database.

Higher education qualifications included when creating the database of conservation degrees. Below qualifications are taken from UKGOV (<https://www.gov.uk/what-different-qualification-levels-mean/list-of-qualification-levels>) and TESQA (<https://www.teqsa.gov.au/australian-qualifications-framework>). Apprenticeships and NQVs were excluded from the database as they are vocational qualifications. MRes degrees were only included if they included at least three taught modules. Level 8 research focused qualifications – PhDs and DPhils – were excluded.

| UK HE Qualifications | Australian HE Qualifications |
| --- | --- |
| Degree with honours – BA or BSc | Bachelor Degree |
| BSc Top Up | Bachelor Honours degree |
| Graduate certificate | Graduate Certificate |
| Graduate diploma | Graduate Diploma |
| Ordinary degree without honours | Master’s Degree (extended) |
| Integrated master’s degree (e.g MEng) | Master’s Degree (coursework) |
| Master’s degree (e.g MA or MSc) | Master’s Degree (Research) * only if multiple taught conservation modules included |
| Master’s degree (MRes) * only if taught multiple taught conservation modules included |  |
| Postgraduate certificate |  |
| Postgraduate diploma |  |

# Appendix S2. Database of conservation specific degrees.

See separate file named S2.

# Appendix S3. Database of conservation specific modules.

See separate file named S3.

# Appendix S4. Copy of online survey instrument.

See separate file named S4.

# Appendix S5. List of conservation textbooks reviewed to create a list of conservation-specific topics.

The below 12 conservation textbooks were selected as they frequently appeared in conservation degrees’ list of essential or recommended reading. The chapters of each textbook were reviewed to create a total of 19 conservation specific topics that were then included as multiple choice options in the online survey instrument.

1. Primack, R. (2012) A Primer of Conservation Biology (5th edition). Sunderland, MA: Sinauer Associates, Inc. Publishers.
2. Hambler, C. & Canney, S.M. (2013) Conservation. Cambridge University Press.
3. Pullin, A. S. (2002) Conservation Biology. Cambridge: Cambridge University Press.
4. Sodhi, N. S., & Ehrlich, P. R. (Eds.). (2010). Conservation biology for all. Oxford University Press.
5. Van Dyke, F. (2008) Conservation Biology: Foundations, Concepts, Applications. Dordrecht: Springer Netherlands.
6. Groom, M. J. & Carroll, C.R., & Meffe, G.K. (2006) Principles of Conservation Biology.(3rd edition). Sunderland, Mass: Sinauer Associates.
7. Primack, R.B. (2014) Essentials of Conservation Biology (6th edition). Sunderland, Massachusetts: Sinauer Associates.
8. Macdonald, D. W, & Willis, K.J. (2013) Key Topics in Conservation Biology 2. Aufl. Hoboken: Wiley-Blackwell.
9. Sutherland, W.J. (2008). The conservation handbook: research, management and policy. John Wiley & Sons.
10. Hunter, M. L., & Gibbs, J.P. (2007) Fundamentals of Conservation Biology. (3rd edition). Chichester: Wiley.
11. Fryxell, J. M., Sinclair, A.R.E., & Caughley, G. (2014) Wildlife Ecology, Conservation, and Management (3rd edition). Wiley.
12. Hambler, C. (2004) Conservation. Cambridge: Cambridge University Press

# Appendix S6. List of topics and short descriptions for each.

The 19 topics appeared as multiple-choice options in the online survey. The descriptions, shown in the brackets, appeared when respondents hovered their cursor over each topic option. Guidance below the survey questions informed respondents that this extra description information was available. Respondents were also able to specify any topics that they felt were missing from this list of topics in a separate open text survey response.

1. Biodiversity and biogeography (includes definition of biological diversity, patterns and trends of biodiversity loss)
2. Climate change (includes impacts of climate change on biological processes, species, ecosystems and society)
3. Community based conservation (includes definitions of community based conservation and community conservation strategies)
4. Conservation outside of protected areas (includes ex-situ conservation strategies such as zoos, gardens and landscape scale conservation)
5. Definitions and history of conservation (includes teaching on the origins of conservation, goals of conservation and attributes of conservation as a discipline)
6. Ecology of threatened species (includes teaching on population dynamics, effective population sizes and population analysis methods)
7. Ecosystem functioning and dynamics (includes teaching on ecosystem states, ecosystem resistance and resilience)
8. Ecosystem services (includes teaching on the benefits of ecosystems and use of es concept in conservation)
9. Economics in conservation (includes teaching on economic theories, approaches and instruments in conservation)
10. Engaging public in conservation (includes teaching on citizen science, education and fundraising for conservation)
11. Ethics, philosophy and values (includes teaching on different types of values in conservation, ethics and philosophical questions in conservation)
12. Genetics (includes teaching on genetic diversity, genetic management, the importance and use of genetic information)
13. Governance and legislation (includes teaching on policies, legislation and governing bodies in conservation)
14. Invasive species (includes teaching on invasions of exotic species and non-native species management)
15. Protected areas (includes teaching on protect areas management, design, goals and limitations)
16. Restoration and reintroductions (includes teaching on rewilding, reintroductions, and restoration of populations or ecosystems)
17. Setting conservation priorities (includes teaching on evaluation, prioritisation of species and habitats)
18. Sustainable development (includes teaching on the relationship between conservation, development and SDGs)
19. Threats in conservation (includes teaching on habitat degradation, destruction, fragmentation and direct drivers of species loss)

# Appendix S7. Content analysis protocol for online module descriptions and example of coding.

A description of the steps followed as part of the protocol is provided below (A-I). The online description text was reviewed to check for mentions of the 19 conservation-specific topics identified (Appendix S6). The table shows an example of how the spreadsheet was coded to show where module descriptions mentioned conservation-specific topics.

1. Researcher and temporary assistants assigned conservation-specific modules for UK and Australia about which there is no survey response.
2. Each researcher searched the module in the relevant university catalogue
3. Where no online module catalogue was available, a search for the module was conducted on the university webpages and via google.
4. If a publicly accessible online module description was found, it was checked against the following criteria:
   1. Is the description for the academic year 2020-21 (UK) or 2021-22 (Australia)?
   2. Does the description have an overview/content description section?
   3. Does the description have a section on skills/learning outcomes?
5. If the online module description met the above, it was considered a full module description and eligible for review.
6. The online description was then reviewed to see if it covered any of the 19 specified conservation-specific topics (Appendix S6).
7. Where the topic or the terms in the short description were mentioned, a 1 was noted in the spreadsheet under the relevant topic column.
8. Any topics discussed in the online module description that were not easily categorised into the 19 specific topics were noted in an ‘other’ column
9. Regular validation checks were conducted where researchers reviewed each other’s coding, and a sample of module descriptions were compared.

|  |  |  |  | Biodiversity and biogeography | Climate change | Community based conservation | Conservation outside of protected areas | Definitions and history of conservation | Ecology of threatened species |  |
| --- | --- | --- | --- | --- | --- | --- | --- | --- | --- | --- |
| HEI | Module | Full module description available? | URL to module description | Includes definition of biological diversity, patterns, and trends of biodiversity loss | Includes impacts of climate change on biological processes, species, ecosystems and society | Includes definitions of community based conservation and community conservation strategies | Includes ex-situ conservation strategies such as zoos, gardens and landscape scale conservation | Includes teaching on the origins of conservation, goals of conservation and attributes of conservation as a discipline | Includes teaching on population dynamics, effective population sizes and population analysis methods |  |
| University 101 | Module 101 | Yes | [link inserted to URL] | 1 |  | 1 |  |  |  |  |

# Appendix S8. Comparison of survey and content analysis data.

See separate pdf file named S8.

# Appendix S9: Classification of subject-areas into disciplinary categories

To investigate disciplinary breadth, we grouped the subject-area codes into broader disciplinary categories. The sorting of subject-areas into disciplinary categories was guided by the subject groups in the HESA codes specified by the UK Higher Education Statistics Agency.

| Disciplinary category | Subject-areas included in category |
| --- | --- |
| Biological sciences | Agriculture, Biology, Botany, Ecology, Forestry and arboriculture, Genetics, Medicine, Microbiology, Molecular biology, biophysics and biochemistry, Veterinary science, Zoology |
| Social sciences | Anthropology, Development studies, Economics, Human and social geography, Politics, Psychology, Sociology and social policy, |
| Non-biological sciences | Computer sciences, engineering and technology; Mathematical sciences; Physical sciences |
| Humanities | History, Philosophy |
| Law | Law |
| Mass-communication and documentation | Mass communication and documentation |

# Appendix S10: Sorting of ‘other’ disciplinary staff responses into disciplinary categories

Where respondents selected ‘Other’ for the disciplinary category of academic staff, we reviewed each response and sorted each case where possible. The reasons for the final sorting decisions are provided in the table below. Where ‘geography’ was stated in the other text box response, we classed the staff as interdisciplinary unless there was further information to specify whether the academic staff member held specific expertise in human or physical geography. Similar to the sorting of subject-areas, our sorting of ‘other’ responses was guided by the subject group codes used by the UK Higher Education Statistics Agency. Responses that selected the ‘other’ option but provided no text information were not reclassified for analysis but remained as ‘Other’.

| Academic staff ‘other’ text response | Sorted into survey academic staff discipline category | Reason for sorting into given category |
| --- | --- | --- |
| Agricultural sciences | Biological sciences | Agricultural science typically classed as a biological science in higher education |
| Environmental management | Interdisciplinary | Environmental management often defined as interdisciplinary field spanning natural and social sciences. |
| Geography | Interdisciplinary | Without further information on the academic staff and their focus, unable to class as physical or social/human geography – hence classed as interdisciplinary. |
| Earth sciences | Non-biological sciences | Earth sciences best classed under physical sciences in HESA coding. |
| Law | Created new ‘law’ category. | Law classed separately as not listed under social sciences or humanities in HESA subject codes system basing categories on. |
| Ecologist | Biological sciences | Ecology typically classed under biological sciences |
| Geosciences | Interdisciplinary | Without further information, unable to ascertain which aspect of geosciences and therefore classed as interdisciplinary given it can span both natural and social sciences. |
| Psychology | Social sciences | Psychology often referred to as social science in conservation literature and under social sciences in HESA codes if includes some social science dimension. |

# Appendix S11. Code used to fit generalized linear mixed effect models in RStudio.

Code used to make model for disciplinary categories:

m_dis <- glmer(presence ~ 1 + exclusiveto_pg + country + new.department +

mixstaffdis + discip + (1|module.uid),

family = binomial, data = d_dis,

nAGQ = 0, control = glmerControl(optimizer = "nloptwrap"))

Code used to make model for topics:

m_top <- glmer(presence ~ 1 + exclusiveto_pg + country + new.department +

mixstaffdis + (1|topic) + (1|module.uid),

family = binomial, data = d_top,

nAGQ=0,control=glmerControl(optimizer = "nloptwrap"))

# Appendix S12: Likert responses to interdisciplinary statements in final section of the online survey instrument.

1. UK online survey instrument interdisciplinary Likert responses.

The plot below includes all conservation specific module survey responses, with any survey responses that did not answer a Likert statement removed (UK module n = 109).

1. Australia online survey instrument interdisciplinary Likert responses.

The plot below includes all Australian conservation specific module survey responses, with any survey responses that did not answer a Likert statement removed (n = 29).

#

# Appendix S13. Boxplot of the frequency of subject areas covered by conservation specific modules.

We found a variation in the number of subject-areas covered when looking across the conservation specific modules, using the survey response data (UK = 117, Australia = 29). The boxplot below indicates the average number subject-areas covered in each type of conservation specific module (country and education level), as well as the total range represented in the sample of survey responses for conservation specific modules.

# Appendix 14: Topic frequencies split by survey response and content analysis data.

The below pyramid plots show the percentage of topics that appeared in data for conservation specific modules collected through the survey instrument (UK = 117, Australia = 29) and through the content analysis of online module descriptions (UK = 159, Australia = 63).

# Appendix S15. Boxplot of number of topics covered in conservation specific modules, grouped by country and education level.

Boxplot uses both survey and content analysis data for conservation specific modules.

We found a variation in the number of topics covered when looking across the conservation specific modules, using a combination of data from the survey instrument and content analysis of online module descriptions (UK = 276, Australia = 92). The boxplot below indicates the average number topics covered in each type of conservation specific module (country and education level), as well as the total range represented in the sample of conservation specific modules (using a combination of survey and content analysis data).

# Appendix S16. Conservation degrees including at least one core module on a topic option.

The pyramid plot below shows the percentage of conservation degrees we identified that included at least one core module on each of the topics options. The grey bars are percentages for the postgraduate degrees (calculated out of a total of 30 UK postgraduate and 11 Australian postgraduate degrees). The blue bars show percentages for undergraduate degrees (calculated out of a total of 12 UK undergraduate degrees and 9 Australian undergraduate degrees).

# Appendix S17: Percentage of conservation specific modules belonging to each type of department.

The below bar graph indicates the percentage of conservation specific modules that belonged to each type of disciplinary department. As described in the manuscript, biological and non-biological sciences responses were combined into a new category of ‘Natural Sciences’. Any ‘other’ responses to the survey department question were sorted on a case by case basis, as described in the manuscript. Analysis used solely survey response data for conservation specific modules (UK n = 117, Australia n = 29)

# Appendix S18: Percentage of conservation specific modules including staff from each disciplinary category.

The below bar graph shows the percentage of conservation specific modules that included staff from the different disciplinary categories provided in the survey. This analysis used solely survey response data for conservation specific modules (UK n = 117, Australia n = 29). Survey respondents were able to select multiple options to indicate the different disciplinary categories represented by the academic staff in their module. Any ‘other’ staff responses were sorted on a case by case basis, as described in appendix S10.

# Appendix S19. Percentage of modules covering biological or social sciences, and whether they include the corresponding disciplinary staff expertise.

The below plot indicates the percentage of conservation specific modules that included teaching on biological science subject-areas and social sciences subject-areas. The teach biological sciences/social sciences with or without bars indicate what percentage of modules covering biological sciences or social sciences subject-areas include a member of academic staff from the corresponding disciplinary category. We find that although 60% of conservation specific modules in our survey response dataset included teaching on social sciences subject-areas, just 31% of those modules included an academic staff member from the social sciences. This is in contrast to the biological sciences. This analysis used solely survey response data for conservation specific modules (UK n = 117, Australia n = 29).
