# Supplementary material for: Assessing the breadth and multidisciplinarity of the conservation curriculum in the United Kingdom and Australia
Source: Bioscience. 2024 Aug 1;74(9):652–62. doi: 10.1093/biosci/biae059 (PMC11480662; doi:10.1093/biosci/biae059)
Supplement: biae059_Supplemental_Files [file biae059_supplemental_files.zip › Survey_S4.pdf]

Conservation Education Module Survey

Survey information and privacy statement
About the survey

This survey is part of a PhD research project investigating the role of higher education in shaping the views and skills of future conservationists. This survey aims to collect information on what is being taught in undergraduate and postgraduate conservation modules. The survey will ask questions about the topics, skills and teaching methods being covered in your conservation-related module. The final section of the survey includes questions on interdisciplinarity and relates to later stages of the PhD project which will explore the role of interdisciplinarity in conservation higher education.

Please complete this survey if you:
- lead a module that is offered in a conservation degree programme
OR
- lead a module with 'conservation' in the title (even if it offered outside of a conservation degree programme)

Please complete the survey prospectively for modules that are due to run in the next academic year (2020-2021).

The survey should take 5-10 minutes to complete for a module.

We aim for this survey to be completed by module co-ordinators. If you are a module co-ordinator for multiple conservation-related modules, we would really appreciate it if you could fill complete the survey for each of the modules separately.

Ethics and data management

This project has been approved by the research ethics committee at [removed to maintain anonymity] and is GDPR compliant. Your survey responses will be used as part of a PhD research project on higher education and conservation and may form the basis of peer-reviewed academic publications. All of the data gathered will be stored securely on [removed to maintain anonymity] servers and will solely be used for academic research. An anonymised version of the dataset will be made available to the funders of the research and may be shared with other researchers involved in the PhD project. The anonymised dataset may also be included as supporting material for any related peer-reviewed academic publications. Anonymised datasets will be stored for 10 years (the retention period that the funding body, [removed to maintain anonymity], requires). Your individual survey responses will not be identifiable in the PhD thesis or any related publications. You are welcome to contact the researcher (removed to maintain anonymity), prior to the lodging of the dataset, if you wish for your data to be removed from the

dataset. If you are worried about this research, or if you are concerned about how it is being conducted, you can contact the [removed to maintain anonymity]

Project updates

If you would like to receive a summary of the findings from this survey, there will be an option to leave your email address at the end of the survey. This information will not be passed on to any third parties and will only be used to contact you about this research project.

Contact information

If you have any questions, please contact [removed to maintain anonymity].

By clicking the next button you provide consent to the above:

Page Break

Module Information

Q1 What is the name of the conservation-related module you teach/organise?

____

Q2 What is the module code, if applicable? *Please enter the code that your institution uses for this module.*

____

Q3 What higher education institution is the module taught at?

____

Q4 During the academic year 2020-2021, when does the module start? Enter a date:

____

Q5 During the academic year 2020-2021, when does the module finish? Enter a date:

____

Q6
At your institution, what academic department does the module belong to?

Please select the most appropriate option that best reflects the department the module belongs to at your higher education institution.

- ◯ Biological sciences (e.g. zoology, plant sciences) (1)
- ◯ Social sciences (e.g. economics, human geography, political sciences) (2)
- ◯ Non biological sciences (e.g. chemistry, maths, engineering) (5)
- ◯ Humanities (e.g. literature, history, philosophy, languages) (3)
- ◯ Interdisciplinary (e.g. a department of interdisciplinary studies) (4)
- ◯ Other (7) ____

Q7 How long has the module has been running for?

- ◯ 0-2 years (1)
- ◯ 3-5 years (2)
- ◯ 6-8 years (3)
- ◯ 9+ years (4)

Q8 Does the module teach undergraduate or postgraduate students?

- ◯ Undergraduates (1)
- ◯ Postgraduates (such as MSc and MRes students) (2)
- ◯ Undergraduates and postgraduates (3)

Q9 Is this module compulsory or optional?

- ○ Compulsory (1)
- ○ Optional (2)
- ○ Depends on student degree path (3)
- ○ Unsure (4)

Q10 Can the module be taken by students studying a non-conservation specific degree?

By non-conservation degree we mean any degree that does not have 'conservation' in the title (such as Biology BSc or Environmental Science MSc).

- ○ Yes (1)
- ○ No (2)

Q11 How many members of academic staff are involved in the teaching of the module?

▼ 1 (1) ... 10+ (10)

Q12 What academic disciplines do the module staff belong to? *Please select all that apply.*

- ▢ Biological sciences (e.g. zoology, plant sciences) (1)
- ▢ Social sciences (e.g. economics, human geography, political sciences) (2)
- ▢ Non-biological natural sciences (e.g. chemistry, maths, engineering) (3)
- ▢ Humanities (e.g. literature, history, philosophy, languages) (4)
- ▢ Interdisciplinary (a combination of at least 2 natural sciences, social sciences and humanities) (5)
- ▢ Other (6) ____

Q13 Does the module include teaching from any individuals working primarily outside academia?

- ○ Yes (1)
- ○ No (2)

Display This Question:

If Does the module include teaching from any individuals working primarily outside academia? = Yes

Q12a In what capacity are the individuals from outside academia involved in the module? *If 'Other' please specify.*

- ☐ As guest lecturers/teachers (1)
- ☐ As research partners (2)
- ☐ As supervisors for research project(s) (3)
- ☐ As student internship/placement supervisors (4)
- ☐ Other (5) ____

Display This Question:

If Does the module include teaching from any individuals working primarily outside academia? = Yes

Q13b Which of the following are the non-academic individuals from? *Please select all that apply.*

- ☐ Non Governmental Organisations (1)
- ☐ Governmental organisations (2)
- ☐ Private sector organisations (3)
- ☐ Other (4) ____

Page Break

Module content

Q14 Please select all of the following subject areas that the module includes teaching on:

Please note this question relates to subject areas and we will ask you separately about specific conservation topics in a subsequent question.

- [ ] Agriculture (11)
- [ ] Anthropology (20)
- [ ] Biology (2)
- [ ] Botany (5)
- [ ] Computer sciences, engineering and technology (16)
- [ ] Development studies (22)
- [ ] Ecology (3)
- [ ] Economics (17)
- [ ] Forestry and arboriculture (13)
- [ ] Genetics (6)
- [ ] History (25)
- [ ] Human and social geography (21)
- [ ] Law (23)
- [ ] Mass communication and documentation (24)
- [ ] Mathematical sciences (includes mathematics and statistics) (15)
- [ ] Medicine (1)
- [ ] Microbiology (7)

☐ Molecular biology, biophysics and biochemistry (8)

☐ Philosophy (26)

☐ Physical sciences (includes chemistry, physics and physical geographic sciences) (14)

☐ Politics (18)

☐ Psychology (9)

☐ Sociology and social policy (19)

☐ Veterinary science (10)

☐ Zoology (4)

☐ Other (27) ____

Q15 Please select all of the following conservation specific topics that are covered in the module:

Please note this question relates to specific conservation subject matter and not skills or methods teaching - there will be a question on skills/methods teaching later in the survey.

**** We are aware some topics may fall under more than one of these broader categories.***

Please hover over the text options for more information about the topics included in each option

- ☐ Biodiversity and biogeography (3)
- ☐ Climate change (14)
- ☐ Community based conservation (17)
- ☐ Conservation outside of protected areas (7)
- ☐ Definitions and history of conservation (1)
- ☐ Ecology of threatened species (5)
- ☐ Ecosystem functioning and dynamics (19)
- ☐ Ecosystem services (20)
- ☐ Economics in conservation (16)
- ☐ Engaging public in conservation (8)
- ☐ Ethics, philosophy and values (2)
- ☐ Genetics (15)
- ☐ Governance and legislation (11)
- ☐ Invasive species (13)
- ☐ Protected areas (6)
- ☐ Restoration and reintroductions (12)
- ☐ Setting conservation priorities (10)

☐ Sustainable development (9)

☐ Threats in conservation (4)

Q16 Are there any topics taught in your module that you feel are missing from the options in the previous question? If yes, please state the topics in the box below:

____

____

____

____

____

Q17 Does your module focus on one or more specific biomes?

○ Yes (1)

○ No (2)

Display This Question:

If Does your module focus on one or more specific biomes? = Yes

Q17a Please select all biomes that the module focuses on:

- ▢ Forests (1)
- ▢ Freshwater (3)
- ▢ Marine (4)
- ▢ Savanna/grasslands (2)
- ▢ Tundra (5)
- ▢ Other (7) ____

Q18 Does your module focus on specific taxonomic groups, rather than conservation in general?

- ◯ Yes - there is a focus on specific taxonomic groups (1)
- ◯ No - there is no focus on specific taxonomic groups (2)

Display This Question:

If Does your module focus on specific taxonomic groups, rather than conservation in general? = Yes - there is a focus on specific taxonomic groups

Q18a Please select all of the below taxonomic groups that the module focuses on:

- ▢ Amphibians (5)
- ▢ Birds (2)
- ▢ Fish (3)
- ▢ Invertebrates (6)
- ▢ Mammals (1)
- ▢ Plants (7)
- ▢ Reptiles (4)
- ▢ Other (8) ____

Q19 Does your module focus on specific geographic zones, rather than conservation in general?

- ◯ Yes - the module has a focus on specific geographic zones (1)
- ◯ No - the module does not focus on specific geographic zones (2)

Display This Question:

If Does your module focus on specific geographic zones, rather than conservation in general? = Yes - the module has a focus on specific geographic zones

Q19a Please select all of the geographic zones that the module focuses on:

- ▢ Polar (3)
- ▢ Tropical (1)
- ▢ Temperate (2)
- ▢ Sub-tropical (6)
- ▢ Other (5) ____

Page Break

Q20 Please select all of the following research methods that are taught in the module:

**** You can hover over the text options for more information***

- [ ] Qualitative social science research methods (1)
- [ ] Quantitative social science research methods (2)
- [ ] Field based methods (3)
- [ ] Lab based methods (4)
- [ ] Statistics and modelling (5)
- [ ] Remote sensing and GIS methods (6)
- [ ] Other (8) ____

Q20 Please select all of the below skills that the module aims to develop:

*** You can hover over the first two text options for more information**

- ▢ Specific disciplinary skills (1)
- ▢ General disciplinary skills (2)
- ▢ Field skills (3)
- ▢ Written communication (4)
- ▢ Technical and information technology skills (5)
- ▢ Oral communication (6)
- ▢ Project management (7)
- ▢ Program leadership (8)
- ▢ Networking skills (9)
- ▢ Conflict resolution and negotiation skills (16)
- ▢ Inter- and multidisciplinary skills (11)
- ▢ Interpersonal skills (14)
- ▢ Outreach communication (10)
- ▢ Multitasking and prioritisation skills (13)
- ▢ Personnel leadership (15)
- ▢ Cultural and international experience (12)
- ▢ Other (17) ____

Page Break

Teaching methods

Q21 Please estimate what percentage of the module teaching time is spent using the following teaching methods?

Please drag circle on each bar to change the percentage. You can keep a circle at 0 if applicable. Please ensure that the total in the bottom right adds up to 100%.

*** You can hover over each text option for examples of teaching activities**
_______ Practical work (1)
_______ Teacher led activities (2)
_______ Student led activities (4)
_______ Applied work with practitioners (5)
_______ Other (15)

Q22 What modes of summative assessment are used in the module? *Please select all that apply.*

Summative assessment refers to assessments used to evaluate student learning at the end of a defined teaching period

- ☐ Written exam (1)
- ☐ Scientific report (2)
- ☐ Oral presentation (3)
- ☐ Group presentation (4)
- ☐ Group report (9)
- ☐ Essay (5)
- ☐ Creative assessment (e.g. film, podcast) (6)
- ☐ Policy briefing/white paper report (7)
- ☐ Other (8) ____

Q58
Please estimate what percentage of the module will be delivered online in the next academic year (2020-2021).

Please drag the circle to change the percentage. You can keep the circle at 0 if applicable.

	0	10	20	30	40	50	60	70	80	90	100
4 ()											

Page Break

Interdisciplinarity

Q23 Does your module specifically aim to teach students how to address interdisciplinary topics?

○ Yes (1)

○ No (2)

Q24 What does the term 'interdisciplinary teaching' mean to you?

____

____

____

____

____

Q25 Please indicate to what extent you agree with the following statements:

	Strongly disagree (1)	Disagree (2)	Neither agree nor disagree (3)	Agree (4)	Strongly agree (5)
Students become interdisciplinary thinkers by taking a diverse range of disciplinary courses (2)	o	o	o	o	o
It is important to train conservation students as interdisciplinary thinkers (1)	o	o	o	o	o
An individual lecturer can teach interdisciplinary materials from different disciplines (3)	o	o	o	o	o
Interdisciplinary teaching brings together individuals to share insights from different disciplinary courses (4)	o	o	o	o	o
Interdisciplinary teaching waters down training and students should focus on becoming experts in their discipline (9)	o	o	o	o	o

The natural sciences should be the primary focus of conservation training (7)	○	○	○	○	○
Interdisciplinary teaching works between disciplines and attempts to dissolve boundaries (5)	○	○	○	○	○
An interdisciplinary course integrates material from multiple disciplines, addressing epistemological and methodological understandings (6)	○	○	○	○	○
Solving conservation problems requires people from different academic disciplines to work together (11)	○	○	○	○	○
The field of conservation needs students who understand the natural and social sciences. (8)	○	○	○	○	○

Solving conservation problems requires interdisciplinary thinkers. (10)	○	○	○	○	○

Page Break

Q26 If you would like to be contacted with a summary of the survey findings, please leave your email address in the box below:

____

End of Block: Default Question Block
